# Exploring associations between area deprivation index, income, and loneliness among cancer caregivers

**DOI:** 10.1007/s00520-026-10578-1

**Published:** 2026-03-18

**Authors:** Kyle Pitzer, JoAnn Jabbari, George Demiris, Ramy Sedhom, Jacquelyn J. Benson, Debra Parker Oliver, Karla T. Washington

**Affiliations:** 1 School of Medicine, Washington University in St. Louis, 660 S. Euclid Ave., St. Louis, MO 63110-1010 USA; 2 Goldfarb School of Nursing at Barnes-Jewish College, 4483 Duncan Ave., St. Louis, MO 63110 USA; 3 Department of Biostatistics, Epidemiology and Informatics, University of Pennsylvania Perelman School of Medicine, 3400 Civic Center Blvd., Philadelphia, PA 19104 USA; 4 Department of Biobehavioral Health Sciences, University of Pennsylvania School of Nursing, 418 Curie Blvd, Philadelphia, PA 19104 USA; 5 Department of Medicine, University of Pennsylvania Perelman School of Medicine, 3400 Civic Center Blvd, Philadelphia, PA 19104 USA

**Keywords:** Cancer, Caregivers, Area deprivation, Household income, Loneliness

## Abstract

**Purpose:**

Caregivers of patients diagnosed with cancer experience stress associated with their roles and responsibilities as a caregiver. These stressors contribute to psychosocial issues among this population and many caregivers experience loneliness. A caregiver’s neighborhood context and financial circumstances could be associated with loneliness. The current study had two objectives: (1) examine the associations among area deprivation index, household income, and loneliness for caregivers of patients with cancer and (2) identify potential interaction effects between area deprivation index and household income on loneliness.

**Methods:**

Demographic data and baseline surveys for our study were derived from a larger clinical trial of caregivers of patients with cancer receiving outpatient palliative care. Participants were geocoded to determine their census block group and corresponding area deprivation index percentile. Linear models were estimated to assess associations among area deprivation index, household income, and loneliness, and models were subsequently adjusted for several demographic and caregiving context covariates.

**Results:**

The results showed that there was no significant association between area deprivation index and loneliness but did indicate an association between household income and loneliness: caregivers with annual household incomes less than $70,000 experienced greater loneliness on average than those with annual household incomes greater than $70,000. There was no detectable interaction effect of area deprivation index and household income on loneliness.

**Conclusion:**

These results indicate that financial and income data should be considered when developing clinical practices or infrastructure to ameliorate loneliness in caregivers of patients with cancer.

## Introduction

Informal caregivers of family members or friends diagnosed with cancer experience many stressors associated with care provision, communication with healthcare providers, and family decision-making about care plans [1–3]. These stressors can lead to increased risk of loneliness as well as other psychosocial outcomes. A recent longitudinal study reported that approximately 30% of cancer caregivers experience high levels of loneliness, and these numbers remain consistent over time [4]. Loneliness among caregivers is associated with adverse outcomes such as depression, anxiety, sleep disorders, frailty, and poorer perceived health [5, 6]. Caregivers also encounter significant financial stressors such as financial toxicity, defined as objective and subjective distress due to the high cost of cancer treatment. A recent study reported that 89% of cancer caregivers are concerned about their financial futures [7]. Approximately 33% of caregivers incur debt to provide care, and 24% report adverse economic situations [8]. Although studies on financial toxicity among cancer caregivers are relatively limited compared to patients, 44% and 57% of caregivers in two recent studies reported financial strain or toxicity, which is similar to the 49% of patients reporting financial burden in a meta-analysis of 25 studies [9–11]. Recent research has also shown relationships between financial toxicity and loneliness in cancer patients, specifically that loneliness partially mediated the relationship between financial burden and quality of life [12]. Given the close tie between patients and their caregivers, these relationships are also important to explore for caregivers.

Further, the socioeconomic area where caregivers live can compound the challenges of providing care. Research examining the association between where caregivers of patients with serious illnesses, such as dementia and cancer, reside and psychosocial outcomes reported that neighborhood income and area deprivation index (ADI) were associated with outcomes such as distress and anxiety [13, 14]. Although there is emerging evidence that neighborhood environments affect psychosocial outcomes among caregivers of patients diagnosed with cancer, there is limited research on the effects of neighborhood environments on loneliness specifically among caregivers of patients with cancer.

Given previous work on loneliness, income, and neighborhood environment among cancer patients and their caregivers, we posit that these specific components may be related in specific ways among cancer caregivers that have not yet been elucidated by current literature. This study explored whether household income and ADI are associated with loneliness among caregivers of patients with cancer, and whether loneliness and ADI are differentially associated by household income. The results of this study contribute to the understanding of individual- and community-level factors that influence the experience of loneliness among caregivers and identify intervention targets to mitigate the adverse effects of loneliness.

### Theoretical foundations

**Fig. 1:**
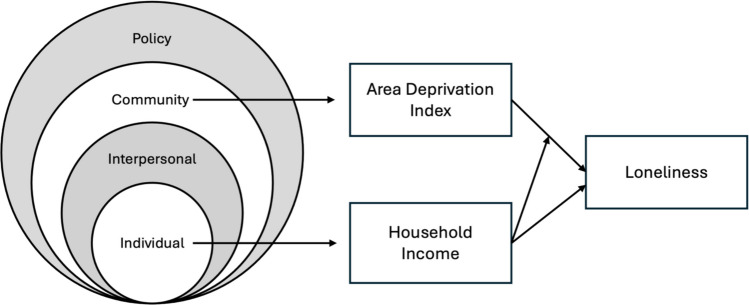
Visual representation of the social ecological framework and key variable associations

This study is informed by the Social Ecological Model (SEM) and the theory and research on the impact of neighborhood context on a variety of outcomes. The SEM is derived from Bronfenbrenner’s Ecological Systems Theory [15]. SEM posits that individuals exist within a series of interconnected levels, including individual, interpersonal, community, and policy levels, and each level can affect the development and well-being of individuals. Thus, SEM is well-suited for evaluating potential links between individual characteristics, community conditions, and loneliness. A visual representation of the potential relationships between the key variables in our study that relate to SEM is shown in Fig. 1. In addition to this broader framework, decades of research indicate that neighborhood environments are associated with multiple bundles of outcomes. Wilson referred to these as “concentration effects” and concluded that these effects were primarily from living in a neighborhood with high poverty, superseding the effects of individual circumstances [16]. Numerous studies have examined physical and social characteristics of neighborhoods and found effects on the physical and mental health outcomes of residents [17, 18].

Our study utilizes these theoretical foundations to investigate factors that could be extended to the study of financial toxicity in cancer care. Previous studies report that financial toxicity is associated with patient quality of life and mortality, and caregiver health-related quality of life, family relationships, and work situations [19–21]. Consideration of the combined effects of living environment and individual circumstances could generate a clearer picture of a caregiver’s level of care-related financial burden and potential consequences. Subsequently, identifying these socioeconomic factors and modifying clinical practices and infrastructure could improve caregiver levels of financial toxicity as well as related outcomes such as loneliness.

### Current research on neighborhood context, income, and loneliness

Studies have demonstrated that individual and neighborhood-level socioeconomic factors can affect psychological distress and loneliness among caregivers. Caregivers who experience financial strain are more prone to loneliness [22]. A recent study reported that family caregivers with incomes between 100 and 199% of federal poverty guidelines were significantly more likely to experience loneliness than those with other income levels [23]. Another study of cancer caregivers reported that those below the median sample income ($75,000) reported higher rates of loneliness among other psychosocial outcomes [24].

Several studies have also shown that neighborhood context was associated with psychosocial outcomes for caregivers. Caregivers of patients with Alzheimer’s disease experienced higher distress in neighborhoods with lower median household incomes than those in high-income neighborhoods [13]. By contrast, caregivers living in neighborhoods designated as environmental justice or medically underserved areas had lower rates of depression, suggesting that household income alone could be linked to depression and distress [25]. Another recent study of caregivers for patients diagnosed with cancer reported that ADI was differentially associated with anxiety depending on household income: caregivers with lower household incomes experienced higher anxiety as ADI increased [14]. However, few studies have examined neighborhood context and loneliness among caregivers for patients with cancer.

The results of many studies indicate that neighborhood context is associated with loneliness. Studies across multiple countries report that neighborhood infrastructure and social environment can influence loneliness among residents [26]. For example, studies have found associations between neighborhood characteristics, such as the built environment, higher unemployment, and lower income, and loneliness [27, 28]. Studies have also found that social cohesion and neighborhood attachment are also associated with loneliness [29, 30]. Although caregivers were not the focus of these studies, they are a special subgroup of these larger populations, suggesting that neighborhood infrastructure and social environment could influence the psychosocial outcomes experienced by caregivers.

## Methods

### Sample

Data for this secondary analysis were retrieved from Problem-Solving Therapy for Cancer Caregivers: A Randomized Clinical Trial in Outpatient Palliative Care (R01CA258311, ClinicalTrials.gov ID: NCT04867122, Principal Investigator: [AUTHOR]), which is a multisite, randomized clinical trial of a supportive intervention for family caregivers of patients with cancer receiving outpatient palliative care. The study population was recruited from outpatient palliative oncology clinics affiliated with three academic health systems. These included a large metropolitan health system in Missouri serving predominantly urban and suburban patients and their families; a mid-sized Missouri health system with extensive outreach to rural communities; and a large metropolitan health system in Pennsylvania that comprises both a specialized geriatric oncology clinic and clinics serving broader palliative oncology populations. We used data from the baseline survey only (before beginning the intervention) to avoid intervention effects on the outcome. A total of *n* = 449 caregivers completed the baseline survey at the time of our analysis; however, we used complete case analysis, which resulted in a sample size of *n* = 376 for cases with valid data on all model variables. All participants provided informed consent before participating in the study.

### Measures and variables

#### UCLA loneliness scale

Our primary outcome was loneliness as measured by the UCLA 3-Item Loneliness Scale [31]. This survey is assessed by summing the Likert-scale scores for the three survey items, which measure how often respondents experience feelings related to loneliness [ranging from 1 (*hardly ever*) to 3 (*often*)], with total scores ranging from 3 to 9. Higher scores indicate greater levels of loneliness. This measure showed good internal consistency reliability in our study sample (Cronbach’s *α* = 0.83).

#### Area deprivation index

The first predictor of interest was ADI percentile score. We geocoded participants to assign a Federal Information Processing Standard (FIPS) code, which identifies unique geographic areas [32]. Then, we matched census block ADI values using the Neighborhood Atlas developed at the University of Wisconsin [33, 34]. ADI is a widely used measure of percentile ranking at the state and national levels based on four domains: income, education, employment, and housing quality [33, 34]. We used the national ADI percentiles because participants were from multiple states. ADI national percentiles range from 0 to 100, with higher percentiles mapping to areas with greater deprivation. ADI percentiles for participants who lived in census blocks that were classified as group quarters, those with questionable data integrity, or those assigned based on post office boxes were excluded from our analysis.

#### Household income

The second predictor of interest was household income, which was recorded at baseline as a four-category variable: < $20,000, $20,000–$40,000, $40,000–$70,000, and > $70,000. Due to the small proportions of participants in the annual household income groups less than $70,000 (using non-missing data)—7% had < $20,000, 12% had $20,000–$40,000, 23% had $40,000–$70,000—we created a binary variable for annual household income with values of < $70,000 or > $70,000.

#### Covariates

We adjusted for six demographic covariates in our models that were considered potentially important: age; race (White, Black, or Other); ethnicity (non-Hispanic/Latino or Hispanic/Latino); gender (male, female, or other, although no responses of “other” were recorded in our analytic sample); marital status (single, married, in a committed partnership, divorced or separated, widowed, or other); and caregiver relationship to patient (adult child, spouse/partner, other family member, or non-relative). Participants self-reported all data via the baseline survey.

### Data diagnostics

Descriptive statistics indicated that cases with missing data resulted in a sample decrease of ~16% in our models. Missingness on individual variables was < 4% for all variables except for income, which was 6.9%. We were unable to conclude that data were missing completely at random (MCAR) using Little’s test, *χ*^2^(125) = 155.32, *p* = 0.03. We then examined differences between missing and non-missing data on the outcome measure across all explanatory variables using chi-square tests. The results suggested that the missingness in our outcome was likely missing at random (MAR); however, there was a significant difference in age between participants with missing and non-missing data on loneliness. Those with missing data were slightly younger (m = 47.4, sd = 14.7) on average than those with valid data (m = 55.6, sd = 15.4); however, only 5.1% (23) of composite loneliness values were missing from the completed baseline surveys (*n* = 449).

Based on the findings that our missing data were likely MAR, the low amount of missing data by variable (< 7%), some missing data not being “truly missing” (i.e., responses of “don’t know/unsure” or “prefer not to answer” rather than no response), and the collection of data by research specialists within a strictly monitored randomized controlled trial, we did not consider there to be a serious risk of bias and proceeded with listwise deletion and complete case analysis (*n* = 376). We also examined model assumptions using a scatterplot for residuals and fitted values, a normal Q-Q plot, a scale-location plot, and variance inflation factors. In examining our assumptions, we found evidence of a possible violation of the assumption of homoscedasticity and addressed this in our analysis.

### Analysis plan

Before estimating our linear models, we examined our sample and model variables using descriptive statistics of mean, standard deviation, median and range for continuous variables, and frequencies for categorical variables. We then estimated our linear models using a block-wise approach. We estimated two bivariate models in which loneliness was separately regressed on ADI and household income. Model 2 included both predictors of interest and our covariates. Model 3 included the interaction term between annual household income and ADI. Due to evidence of a possible violation of our assumption of homoscedasticity, we also estimated our adjusted model using robust standard errors. We used model estimates and significance to make conclusions regarding our research objectives. We also examined overall fit of the model to the data using *F* tests and *R*^2^. We considered two-tailed *p* values < 0.05 as statistical evidence of associations between our explanatory and outcome variables. All analyses were performed in R (version 4.4.0).

## Results

### Sample characteristics

The sample characteristics are presented in Table 1. Most caregivers were married (67%), White (78%), and identified as female (70%), with an average age of 56. Caregivers primarily identified as the spouse/partner of the patient (55%), followed by adult children (23%), family members (18%), and non-relatives (5%). A total of 58% of the sample had an annual household income greater than $70,000, and 42% had an annual household income less than $70,000. The average ADI percentile was 48. The average score for loneliness using the UCLA 3-Item Loneliness Scale was 5.18 (SD = 1.96), which is slightly lower than the median possible score.

**Table 1:** Sample characteristics

	Total sample (*N* = 376)
Age	
Mean (SD)	55.6 (14.9)
Median [min, max]	58.0 [19.0, 95.0]
Race	
White	293 (77.9%)
Black	51 (13.6%)
Other	32 (8.5%)
Gender	
Male	113 (30.1%)
Female	263 (69.9%)
Marital status	
Single, never married	39 (10.4%)
Married	252 (67.0%)
Committed partnership	45 (12.0%)
Divorced or separated	27 (7.2%)
Widowed	9 (2.4%)
Other	4 (1.1%)
Relationship to patient	
Adult child	86 (22.9%)
Spouse/partner	205 (54.5%)
Family member	66 (17.6%)
Non-relative	19 (5.1%)
Household income	
> $70,000	219 (58.2%)
< $70,000	157 (41.8%)
ADI national percentile	
Mean (SD)	47.7 (26.3)
Median [min, max]	44.5 [2.00, 99.0]
UCLA loneliness	
Mean (SD)	5.18 (1.96)
Median [min, max]	5.00 [3.00, 9.00]

### Model results

All model results are presented in Table 2. The bivariate models for ADI and household income detected an association between household income and loneliness (*b* = 0.497, se = 0.204) but did not detect an association between ADI and loneliness. The association between household income and loneliness and the absence of association between ADI and loneliness remained in the adjusted model. The results of our adjusted model indicate that loneliness increased by 0.44 points (*b* = 0.438, se = 0.220) for caregivers with annual household incomes less than $70,000 compared to caregivers with an annual household income greater than $70,000 on average. The adjusted model also indicated that essentially all covariates were associated with loneliness in some way. Female caregivers were lonelier on average than male caregivers and those who were older, Black, married, in a committed partnership, with marital statuses reported as “other”, or non-relatives were less lonely on average compared to their respective reference groups on average.

**Table 2:** Model results for loneliness, area deprivation index, and household income

	ADI model *b* (se)	Income model *b* (se)	Adjusted model *b* (se)	Interaction model *b* (se)	Robust SE model *b* (se)
Intercept	5.058 ***	4.968 ***	6.871 ***	6.835 ***	6.871 ***
	(0.210)	(0.132)	(0.523)	(0.556)	(0.514)
Area deprivation index					
ADI	0.002		0.002	0.002	0.002
	(0.004)		(0.004)	(0.005)	(0.004)
Household income (ref = > $70,000)					
< $70,000		0.497 *	0.438 *	0.518	0.438 *
		(0.204)	(0.220)	(0.471)	(0.216)
Age					
Age			−0.020 *	−0.020 *	−0.020 **
			(0.008)	(0.008)	(0.008)
Race (ref = White)					
Black			−1.200 ***	−1.195 ***	−1.200 ***
			(0.308)	(0.310)	(0.280)
Other race			0.181	0.185	0.181
			(0.357)	(0.357)	(0.374)
Gender (ref = male)					
Female			0.494 *	0.491 *	0.494 *
			(0.221)	(0.221)	(0.232)
Marital status (ref = single, never married)					
Married			−1.299 **	−1.299 **	−1.299 ***
			(0.405)	(0.405)	(0.390)
Committed partnership			−1.573 ***	−1.563 ***	−1.573 ***
			(0.426)	(0.429)	(0.408)
Divorced or separated			−0.617	−0.622	−0.617
			(0.501)	(0.503)	(0.449)
Widowed			−0.139	−0.146	−0.139
			(0.734)	(0.736)	(0.791)
Other marital status			−2.382 *	−2.385 *	−2.382 ***
			(0.990)	(0.991)	(0.565)
Caregiver relationship (ref = adult child)					
Spouse/partner			0.507	0.508	0.507
			(0.296)	(0.296)	(0.279)
Family member			−0.626	−0.624	−0.626 *
			(0.347)	(0.347)	(0.311)
Non-relative			−1.056 *	−1.059 *	−1.056 *
			(0.489)	(0.490)	(0.430)
Interaction					
ADI* < $70,000				−0.002	
				(0.008)	
*N*	376	376	376	376	376
*R*^2^	0.001	0.016	0.160	0.160	0.160
Adjusted* R*^2^	−0.002	0.013	0.127	0.125	0.127
*F*	0.410	5.931	4.906	4.569	5.868
*P*	0.522	0.015	< 0.001	< 0.001	< 0.001
AIC	1579.197	1573.693	1540.121	1542.082	

***p < 0.001; **p < 0.01; *p < 0.05

We also estimated a model with an interaction term for ADI and annual household income but did not detect evidence of an interaction effect on loneliness. This model also did not improve model fit; therefore, we retained our adjusted model. Lastly, we estimated our adjusted model with robust standard errors because our assumption of homoscedasticity may have been violated. The results were essentially identical in adjusted models with or without robust standard errors. The *F* test for our adjusted model indicated that it had a good fit to the data (*F* = 4.906, *p* < 0.001). The full model explained approximately 16% of the variation in loneliness (*R*^2^ = 0.160), whereas the significant variables alone explained approximately 13% of this variation (adjusted *R*^2^ = 0.127).

## Discussion

Our results indicated that ADI was not associated with loneliness and did not have an interaction with household income. However, household income alone was associated with loneliness. The literature on the relationship between neighborhood environment and various psychosocial outcomes among caregivers and other populations suggested that we would find an association with loneliness in our sample. However, our results regarding loneliness did not align with studies on other psychosocial outcomes [13, 14, 25]. We expected to detect this association due to the importance of social networks and neighborhood attachment in mitigating loneliness [29, 30]. One possible explanation for why this association was not present in our data is that our sample was generally well-resourced (e.g., high income, < 50 ADI) which could have enabled them to circumvent any limitations posed by their immediate surroundings to fulfill their social needs. Another explanation could be that area deprivation simply may not be a good proxy or representation of the social capital derived from one’s immediate neighborhood, and some direct measurement of social capital is required. Despite this divergence, the result that lower household income was associated with greater loneliness is consistent with much of the literature. For example, studies using different metrics for income reported that caregivers with lower incomes report higher loneliness compared to those with higher incomes [23, 24]. Our results add to this literature regarding household income and loneliness and suggest that the effect of ADI on cancer caregiver outcomes needs further research. Household income may be linked to a caregiver’s ability to form new and maintain current social relationships, such as limiting the ability to engage in groups where this formation and maintenance can occur (e.g., clubs, volunteer groups, hobby classes), which could affect feelings of loneliness regardless of ADI.

Our results also suggest a need for more research on caregivers with lower incomes. Although we did detect an association between loneliness and household income, the difference in loneliness was modest for those with lower household income compared to those with higher household income on average. Most of our sample with annual household income below $70,000 had incomes between $40,000 and $69,999. This distribution of caregiver household incomes may have affected the magnitude of our estimates, as we would expect a larger difference in loneliness in light of the evidence that caregivers with lower incomes experience loneliness at a greater rate than those with higher incomes [23, 24]. A research focus on caregivers with lower incomes specifically would provide greater insight into the needs and outcomes for this population of caregivers.

Our study potentially contributes new knowledge to the topic of financial toxicity and associated caregiver outcomes. Our findings regarding household income and loneliness suggest that loneliness could be an outcome of financial toxicity related to caring for a patient diagnosed with cancer. While we were unable to evaluate financial toxicity directly, other studies have reported that lower household incomes and care-related financial toxicity were associated [10, 35]. In addition to the demands of caregiving itself, financial toxicity related to cancer care may limit a caregiver’s participation in social relationships and events, which could then increase their loneliness. This may account for the association we detected between household income and loneliness, suggesting that loneliness is an important outcome to consider when caregivers may have high levels of financial toxicity [5, 6].

Our work has clinical implications for those working with caregivers of patients with cancer. Loneliness could be used as a potential symptom of financial toxicity among caregivers, particularly for those that already have lower household incomes. For example, if a caregiver reports loneliness, the reason for this outcome should be evaluated for financial toxicity and the psychological strain of caregiving, specifically for those with lower household incomes. Interventions that mitigate financial toxicity may alleviate loneliness by providing resources related to the financial burden of cancer care, which subsequently enable caregivers to engage in and maintain social connections with friends and family, and this connection should be explored in future research. Current methods to screen and address financial toxicity include cost-of-care conversations, financial navigation, and direct financial support [36, 37].

This study had four limitations that should be considered. (1) This is a cross-sectional secondary data analysis, which prevents us from identifying causal relationships between ADI, income, and loneliness. (2) Our sample was generally well-resourced, and most caregivers were non-Hispanic White, which limits the generalizability of our results to economically and racially diverse cohorts. (3) Our data were derived from a clinical trial that recruited participants from outpatient palliative care. Thus, our results may not be generalizable to caregivers of patients in other care settings. (4) We did not account for measures of wealth, which carry considerable racial and ethnic disparities. Wealth can provide financial security (e.g., owning a home or possessing emergency savings). These resources cannot be measured only by income. Future studies should utilize longitudinal data for ADI, household income, and loneliness, or a matched control group, and should evaluate associations among these parameters in other cancer care settings and in study samples with greater racial and economic diversity. It will also be important to examine the role of wealth in the associations between ADI, household income, and loneliness in caregivers.

## Conclusion

Our study explored the associations between ADI, household income, and loneliness among caregivers of patients with cancer receiving outpatient palliative care. Our study detected an association between household income and loneliness, but not between ADI and loneliness. These results suggest that caregivers of patients with cancer who have lower household incomes may experience greater levels of loneliness than those with higher household incomes. Financial toxicity related to cancer care may create conditions that exacerbate feelings of loneliness among caregivers. Further research is needed to confirm and expand our results for caregivers with low household incomes. However, our study reveals that caregivers with low household incomes may require specific supports targeting financial hardship and associated outcomes, which could mitigate potential feelings of loneliness. These supports could include cost-of-care conversations, financial navigation, and direct financial assistance. Our results may have clinical applications to reduce adverse outcomes among caregivers of patients with cancer.

## Data Availability

The data that support the findings of this study were obtained from ongoing research. Study participants providing informed consent were not advised that their data would be made publicly available. Deidentified data may be available upon reasonable request from the corresponding author at the completion of the parent study.
